# Editorial: Edible Bird’s Nest—Chemical Composition and Potential Health Efficacy and Risks

**DOI:** 10.3389/fphar.2021.819461

**Published:** 2022-01-28

**Authors:** Geng Li, Ting Hun Lee, Gallant Chan, Karl Wah Keung Tsim

**Affiliations:** ^1^ Laboratory Animal Center, School of Chinese Pharmaceutical Science, Guangzhou University of Chinese Medicine, Guangzhou, China; ^2^ School of Chemical and Energy Engineering, Faculty of Engineering, Universiti Teknologi Malaysia, Johor, Malaysia; ^3^ Division of Life Science and Center for Chinese Medicine, The Hong Kong University of Science and Technology, Hong Kong, China

**Keywords:** edible bird’s nest, chemical composition, potential health efficacy, risk, anti-aging effect

Edible bird’s nest (EBN) is a popular delicacy in the Asian Pacific region: this is a salivary secretion from several *Aerodramus* spp. swiftlets, mainly *Aerodramus fuciphagus*, which originated in Southeast Asian countries, including Indonesia, Malaysia, Thailand, and Vietnam. The historical record of EBN for human consumption can be tracked back to about 1,500 years ago in China. Today, the consumption of EBN as food or as a medicinal product is very common in Asian populations. According to ancient literature, EBN is able to alleviate respiratory health conditions, such as asthma, and is believed to have an impact in skin physiology. Despite the potential functional roles and popularity of this ethnomedicine in Asian cultures, the pharmacological research on EBN is still very limited. These claims of beneficial effects are questionable due to the lack of strong supporting experimental evidence. On the other hand, the production of EBN today is totally depending on swiftlets. The natural habitat of swiftlets is varied according to their surrounding environment, which therefore greatly affects the final product of EBN. Foreign contaminants not related to bioactive proteins or peptides secreted from the swiftlet are frequently identified. The chemical variation, as well as possible contaminants, of EBN is inevitable, leading to possible risks for the consumers. Nevertheless, the scientific community should provide experimental evidence in identifying the chemical composition, pharmacological property, and diversity of contaminants of EBN.

A total of 10 articles involved in the understanding of EBN in terms of chemical composition and functional properties are included in this research topic, including six original articles and four reviews. Seventy-two authors form Malaysia and China participated in this research topic, which reflected that EBN attracted the attention of many scholars.

In the chemical analysis of EBN, Yeo *et al*., in this collection, have summarized the possible contaminants in EBN, which included adulterants, chemicals, and microbials, as well as the current methods in measuring the authenticity and chemical composition. Moreover, the possible sources of contaminants have been proposed to be derived from swiftlet farms, processing, storage, and transportation of EBNs. The design and management of swiftlet houses as well as EBN post-processing could greatly affect the EBN color. The review also provided the variation of chemical composition, as well as beneficial effects, of EBN, which could depend on various factors, such as geographical location, harvesting place, harvesting season, and processing procedure. The authors have proposed that standardized EBN processing methods are required to better preserve the bioactive effects. Besides this, the health concerns of EBN consumption, as caused by adulterant, chemical, and microbial contaminations, should be addressed by strict adherence to standardized guidelines. Several studies demonstrated that the plausible authentication methods could be translated into industrial settings but would require further development in terms of cost-effectiveness and equipment availability.

In the pharmacological analysis of EBN, Chua *et al*. have provided evidence of EBN in suppressing replicated virus within host cells, which therefore led to reduced viral replication, endosomal viral trafficking, intracellular viral autophagy, secretion of pro-inflammatory cytokines, reorganization of the actin cytoskeleton, and increase in lysosomal degradation of viral materials. In addition, the authors have discussed and proposed the potential application of EBN in clinical treatments of influenza A and coronavirus.

Lee *et al*. have reviewed 124 articles from the Web of Science, which consisted of 119 original research articles and five review papers. They reviewed the possible roles of EBN in anti-aging effects, inhibition of influenza virus infection, alternative treatments in athletes and cancer patients, corneal wound healing, stimulation of human adipose-derived stem cells, potentiation of mitogenic responses, epidermal growth factor-like activities, enhancement of bone strength and dermal thickness, eye care, and neuroprotective and antioxidant effects.

On the other hand, Lim *et al*. have provided experimental evidence to support the role of EBN in preventing kidney injury, as induced by gentamicin, in a rat model, suggesting the reno-protective properties of EBN. They found that pre-treatment with 125, 250, and 500 mg/kg EBN has significantly resulted in lesser scores than the model group. However, the protocol that they used in serum renal profiles as a measurement of kidney function will not be a suitable method to assess renal function in such a short duration of an experiment like ours. Thus, they proposed that future similar trials should have a minimum of 14 days post-intervention to have a better assessment of renal recovery.

Rashed *et al*. have found that the cell viability results showed that there are no cytotoxicity effects in all neuroblastoma and epithelial cell lines when exposed to sialic acid at a concentration of 60 μg/ml or below. The Tukey *post-hoc* test also revealed that the number of active mitochondria in SH-SY5Y is significantly higher when induced with sialic acid compared with the control. The number of active mitochondria could be increased by 195% after the treatment of sialic acid, a major active ingredient of EBN, in cultured SH-SY5Y cell lines. Thus, the authors have proposed the possible use of EBN in treating Alzheimer’s disease.

Fan *et al*. have found the protective effect of EBN in dextran sulfate sodium-mediated colitis in mice: this event could be mediated mainly by restoring the balance of T helper 17/regulatory T cells. Moreover, the authors have found that EBN not only restored β-cell function and insulin signaling by attenuation of oxidative stress-mediated chronic inflammation in type 2 diabetic mice but also enhanced the sperm concentration, sperm motility, as well as the levels of FSH and LH. The characterization of EBN through liquid chromatography–mass spectrometry has identified testosterone as one of the peaks. Besides this, amino acids, estradiol, and sialic acid were among the major peaks being identified.

In terms of the cosmetic function of EBN, Lai *et al*. have revealed that the effects of enzymatic digested EBN showed robust skin moisturizing effects as demonstrated in both *in vitro* and *ex vivo* models. In cultured keratinocytes, the expressions of S100-fused type proteins contributing to skin barrier function in the stratum corneum, *e*.*g*., filaggrin and filaggrin-2, were determined in both mRNA and protein levels, which were markedly induced when treated with digested EBN extract. These lines of evidence therefore suggested the water moisturizing effect of EBN in skin function.

In summary, this research topic has a collection of articles on the chemical and pharmacological analyses of EBN. The critical parameters in standardizing EBN during production have been given. The pharmacological functions of EBN includes reno-protection, skin moisturizing, mitochondrial protection, relieving oxidative stress and inflammation, improving type 2 diabetes, and enhancing male reproduction. This comprehensive description of EBN should stimulate the investigation and application of EBN.

